# Case Report: Severe McCune–Albright syndrome presenting with neonatal Cushing syndrome: navigating through clinical obstacles

**DOI:** 10.3389/fendo.2023.1209189

**Published:** 2023-07-25

**Authors:** Yagmur Unsal, Onur Gozmen, İdil Rana User, Hayriye Hızarcıoglu, Bora Gulhan, Saniye Ekinci, Tevfik Karagoz, Z. Alev Ozon, E. Nazlı Gonc

**Affiliations:** ^1^ Department of Pediatrics, Division of Pediatric Endocrinology, Hacettepe University Faculty of Medicine, Ankara, Türkiye; ^2^ Department of Pediatric Surgery, Hacettepe University Faculty of Medicine, Ankara, Türkiye; ^3^ Department of Pediatrics, Division of Pediatric Gastroenterology, Hacettepe University Faculty of Medicine, Ankara, Türkiye; ^4^ Department of Pediatrics, Division of Pediatric Nephrology, Hacettepe University Faculty of Medicine, Ankara, Türkiye; ^5^ Department of Pediatrics, Division of Pediatric Cardiology, Hacettepe University Faculty of Medicine, Ankara, Türkiye

**Keywords:** McCune Albright syndrome, neonatal Cushing syndrome, metyrapone, adrenalectomy, follow-up

## Abstract

**Background:**

Café-au-lait skin macules, Cushing syndrome (CS), hyperthyroidism, and liver and cardiac dysfunction are presenting features of neonatal McCune–Albright syndrome (MAS), CS being the rarest endocrine feature. Although spontaneous resolution of hypercortisolism has been reported, outcome is usually unfavorable. While a unified approach to diagnosis, treatment, and follow-up is lacking, herein successful treatment and long-term follow-up of a rare case is presented.

**Clinical case:**

An 11-day-old girl born small for gestational age presented with deterioration of well-being and weight loss. Large hyperpigmented macules on the trunk, hypertension, hyponatremia, hyperglycemia, and elevated liver enzymes were noted. ACTH-independent CS due to MAS was diagnosed. Although metyrapone (300 mg/m^2^/day) was started on the 25th day, complete remission could not be achieved despite increasing the dose up to 1,850 mg/m^2^/day. At 9 months, right total and left three-quarters adrenalectomy was performed. Cortisol decreased substantially, ACTH remained suppressed, rapid tapering of hydrocortisone to physiological dose was not tolerated, and supraphysiological doses were required for 2 months. *GNAS* analysis from the adrenal tissue showed a pathogenic heterozygous mutation. During 34 months of follow-up, in addition to CS due to MAS, fibrous dysplasia, hypophosphatemic rickets, and peripheral precocious puberty were detected. She is still regularly screened for other endocrinopathies.

**Conclusion:**

Neonatal CS due to MAS is extremely rare. Although there is no specific guideline for diagnosis, treatment, or follow-up, addressing side effects and identifying treatment outcomes will improve quality of life and survival.

## Introduction

McCune–Albright syndrome (MAS) is a rare mosaic disorder of remarkable complexity with an estimated prevalence of 1/100,000 and 1/1,000,000 (1). Timing of postzygotic missense gain of function mutation of *GNAS* encoding stimulatory Gα_s_ determines the extent of tissue involvement, imposing a unique clinical phenotype. Although a combination of two or more classical features, such as fibrous dysplasia of bone (FD), café-au-lait skin macules, and hyperfunctioning endocrinopathies (gonadotropin-independent gonadal function, nonautoimmune hyperthyroidism, growth hormone excess, and neonatal hypercortisolism), are diagnostic, renal, hepatobiliary, and cardiac involvement have also been reported (2–4).

Adrenocorticotropic hormone (ACTH)-independent adrenal Gα_s_ activation results in the rarest endocrine feature of MAS, which almost invariably presents in the neonatal period: Cushing syndrome (CS). Due to greater burden of Gα_s_-mutation-bearing cells, the presence of CS is correlated with increased number of accompanying features of MAS and a poorer outcome. Although there is spontaneous resolution in 33% of cases with neonatal CS, mortality occurs with a high rate of 20% (4).

A dilemma for the clinician is that most publications to date have been case reports, and there is as yet no guideline for diagnosis, treatment, or follow-up. Here, a rare case of severe CS due to MAS, underlining the unique clinical phenotype specific to the neonatal period, is presented. Our goal is to offer a practical approach based on 3 years of clinical experience of this rare disorder that will help navigate challenges during follow-up.

## Case presentation

A baby girl, born small for gestational age with a birthweight of 2,340 g (−2.1 SDS) and a head circumference of 32.6 cm (−1.61 SDS) was admitted to the neonatal intensive care unit in the first day of life for respiratory distress. She was the second child of a healthy non-consanguineous Caucasian couple, born 38 weeks of gestation via cesarean section following an uneventful pregnancy. Alanine aminotransferase [ALT, 2,376 U/L (normal, 0–40)] and aspartate aminotransferase [AST, 875 U/L (normal, 0–40)] were elevated; gamma-glutamyl transferase and bilirubin were normal. Antibiotics were administered intravenously after a diagnosis of possible neonatal sepsis. Respiratory distress resolved, and liver enzymes decreased (ALT, 687 U/L; AST, 108 U/L). As soon as the antimicrobial treatment was completed, she was discharged in the seventh day of life.

She was referred to our center, 4 days later, for failure to thrive (2,315 g), difficulty in feeding, and deterioration of general health. On physical examination, round facies, elongated philtrum and retro-micrognatia, hyperpigmented macules both at the front and back of the trunk and on labia majora, which do not cross midline, and hypertrichosis on the forehead and extremities were noted (**Supplementary Figure S1**). Newborn reflexes were hypoactive, blood pressure was 100/70 mmHg, and second-degree cardiac murmur was also detected. Systems were normal otherwise. Laboratory findings revealed hyponatremia, impaired renal and liver function tests, tubulopathy, and proteinuria, while blood count was normal (hemoglobin, 10.4 g/dl; leukocyte, 25.0 × 10^3^/μl; platelet count, 449×10^3^/μl) (**Table 1**). Hyponatremia resolved with fluid treatment, while liver enzymes, blood urea nitrogen, and creatinine remained elevated. Further endocrine evaluation revealed an elevated serum basal cortisol [225.68 g/dl (N, 6.7–22.6 µg/dL)] and 24-h urinary free cortisol [1,129 μg/day (N, 1.4–20 μg/day)]. Serum cortisol was not suppressed during overnight high-dose dexamethasone suppression test (**Table 2**) (5). Thyroid hormones were consistent with non-thyroidal illness.

**Table 1:** Laboratory investigations on admission, prior to medical treatment (19 days), after medical treatment (6 months), and post-adrenalectomy.

	11-days	19-days	6-months	9 months*	24 months*	34 months*	Normal range
Renal function tests							
**BUN (mg/dl)**	53.3	23.6	8.9	10.2	11.2	12.3	5-18
**Creatinine (mg/dl)**	0.6	0.5	0.2	0.2	0.2	0.27	0.16-0.39
**Sodium (mEq/L)**	110.0	134.0	136.0	138.0	138.0	140	136.0-146.0
**Potassium (mg/dl)**	4.5	2.3	4.6	3.4	4.8	4.1	4.1-5.3
**Renin (pg/ml)**	–	181.2	460.0	10.2	198.0	54.8	1-8.2
**Aldosterone (pg/ml)**	–	160.0	136.2	–	–		35-300
**Protein (spot urine) (mg/dl)**	52.7	–	170.3	100.2	–	–	0-14
**Creatine (spot urine) (mg/dl)**	5.8	–	31.1	16.6	19.6	29.3	30.5-141.4
**Protein/Cre ratio**	9.0		5.8	6.0	–	–	
**Beta-2 microglobulin (spot urine) (ng/ml)**	19428	–	–	–	–	1085	18.8-24.7
Liver function tests							
**ALP (U/L)**	150	156	581	795	1143	1331	100-450
**ALT (U/L)**	539	539	1667	123	56	38	0-40
**AST (U/L)**	134	134	357	75	69	39	0-40
**GGT (U/L)**	420	423	907	746	126	102	0-40
Bone metabolism							
**Calcium (mg/dl)**	10.2	10.1	9.8	8.7	10.3	10.3	8.8-10.9
**Phosphate (mg/dl)**	4.8	4.7	1.8	2.2	4.4	4.9	3.9-7.7
**PTH (pg/ml)**	NA	–	NA	42.2	62	136.7	0-60
**Tp reabsorption (%)**	NA	99	70.0	72.1	–	–	>80.0

BUN, blood urea nitrogen; ALP, alkaline phosphatase; ALT, alanine aminotransferase; AST, aspartate aminotransferase; GGT, gamma-glutamyl transferase; PTH, parathyroid hormone; Tp, tubular phosphate.

* represents post adrenalectomy.NA, not applicable.

**Table 2:** Endocrine evaluation prior to medical treatment (19 days), after medical treatment (6 months), and post-adrenalectomy.

	19-days	6-months	9 months*	24 months*	34 months*	Normal range
**8 AM ACTH (pg/ml)**	10.5	<0.05	<0.05	7.7	5.2	0-46
**8 AM cortisol (μg/dl)**	225.7	35.0	4.4	1.2	0.8	6.7-22.6
**8 AM cortisol (HDDST) (μg/dl)**	102.3	–	–	–	–	<1.8
**Urinary free cortisol, 24-hours (μg/day)**	1129.6	221.2	–	–	–	1.4-20
**DHEA-S (mcg/dL)**	1275.4	2706.9	102.2	3.8	–	43-214
**DHEA (ng/mL)**	21.8	10.1	37.85	–	–	
**Androstenedion (ng/dl)**	2465	4817	88.9	–	–	
**17-hydroxyprogesteron (ng/dl)**	2.4	–	–	–	–	
**TSH (uIU/ml)**	0.72	1.0	0.7	4.04	3.2	0.4-5.3
**Free T4 (pmol/L)**	9.49	15.6	15.7	11.7	16.6	9.7-19.2
**Free T3 (pmol/L)**	3.4	5.4	4,8	NA	9.7	4.3-6.9
**LH (IU/ml)**	1.9	0.2	0.1	0.1	0.1	
**FSH (IU/ml)**	6.5	1	0.8	0.6	6.5	
**Estradiol (pg/ml)**	12.7	12.1	37.5	36.7	<11.8	
**IGF-1 (ng/ml)**	27 (0.6SDS)	–	–	133 (1.7SDS)	54 (-2.2SDS)	
**IGFBP-3 (ng/ml)**	530 (0.7SDS)	–	–	3153 (1.2SDS)	2224 (-0.1SDS)	
**Growth hormone (ng/ml)**	1.2	–	–	2.6	–	1-13.6

ACTH, adrenocorticotropic hormone; HDDST, high-dose dexamethasone suppression test; DHEA-S, dehydroepiandrosterone sulfate; TSH, thyroid-stimulating hormone; LH, luteinizing hormone; FSH, follicle stimulating hormone.

* represents post-adrenalectomy.NA, not applicable.

ACTH-independent CS and café-au-lait spots suggested MAS. Hypercortisolism-related complications emerged. On the 11th day, hyperglycemia (blood glucose, 250 mg/dl) was seen, and it persisted after cessation of intravenous fluids in the exclusively breastfed neonate; thus, 0.5 U subcutaneous neutral protamine Hagedorn insulin (NPH) (three times a day) was initiated on the 16th day of life when blood glucose was 340 mg/dl, and serum insulin was 18.10 μIU/ml. Hypertension (110/90 mmHg) and hypokalemia were triggered by mineralocorticoid action of excessive cortisol on 20^th^ day. Spironolactone (2 mg/kg/day) was started, and nifedipine (0.5 mg/kg/day) was added in order to control blood pressure (**Supplementary Figure S2**). Since immunosuppressive effects of excess cortisol may increase the risk for opportunistic infections, *Pneumocystis jirovecii* prophylaxis was started and live vaccines were postponed.

Features of MAS and accompanying hyperfunctioning endocrinopathies were screened (**Table 2**). On ultrasonography, adrenal glands were hypertrophic; kidneys showed increased parenchymal echogenicity, loss of separation between the cortex and medulla, and enhanced medullary echogenicity; and size and echogenicity of the liver were normal. Magnetic resonance imaging of the abdomen confirmed that adrenal glands were hypertrophic (right and left adrenal gland were 24×22×18 mm and 18×19×20 mm in size, respectively) and lobulated. Echocardiogram revealed left ventricular hypertrophy. Bone survey verified generalized decrease in bone mass and revealed areas of irregular ossification and radiolucency in radius, ulna, and distal tibia, which were interpreted as osteoporosis due to hypercortisolism (**Supplementary Figure S1**).

### Medical treatment

Metyrapone (300 mg/m^2^/day, per oral, in four doses) was started on the 25th day (**Supplementary Figure S2**) (6). Since liver function tests were impaired, metyrapone was preferred over ketoconazole. Soon after metyrapone was started, hyperglycemia and hypertension improved, enabling the discontinuation of insulin and nifedipine. Spironolactone was also gradually tapered and discontinued after 13 days of metyrapone treatment, and she was discharged.

The dose of metyrapone was adjusted frequently, according to clinical findings and serum cortisol levels during regular visits. However, even after gradually increasing metyrapone dose to 1,850 mg/m^2^/day over the course of 6 months, total biochemical suppression of serum cortisol could not be achieved (**Supplementary Figure S3A**), and the patient had progressive loss of bone mineral density, persistent left ventricular hypertrophy, and a lack of catch-up growth. In addition to that, café-au-lait macules became darker, dehydroepiandrosterone sulfate (DHEA-S) gradually increased (**Table 2**), and previously non-existent marked clitoromegaly was noted as a side effect of high-dose metyrapone. She was also prescribed ursodeoxycholic acid (15 mg/kg/day); however, liver enzymes remained high (**Table 1**).

### Right total and left three-quarters adrenalectomy

Right total and left three-quarters adrenalectomy was carried out at 9 months of age in light of the patient’s continued clinical findings of hypercortisolism, the existence of unfavorable prognostic markers (high cortisol levels upon admission and heart and liver problems), and the adverse effects of high-dose metyrapone. The patient was administered 100 mg/m^2^/day glucocorticoids (GC) perioperatively; however, she developed symptoms of adrenal insufficiency. The required GC dose to attain euglycemia, restore general well-being, and resolve adrenal insufficiency was 300 mg/m^2^/day. Fludrocortisone (0.05 mg/day) was also started. Following surgery, supraphysiological doses of GC were required, as she suffered frequent symptoms of adrenal insufficiency (hypoglycemia, malaise, and loss of appetite). GC dose could be tapered very slowly, and a daily dose of 15 mg/m^2^/day could be attained in 2 months.

As liver function tests, serum cortisol levels and left ventricular hypertrophy all improved following adrenalectomy (**Table 1**). Bilateral nodular adrenal hyperplasia was observed in the pathological evaluation of surgical specimen, while the findings of liver wedge biopsy were non-specific (**Supplementary Figure S4**). Sequence analysis of *GNAS* from the surgical sample of adrenal gland revealed a heterozygous, previously described missense mutation in exon 8 (c.2530C>A, p.Arg844Ser), while the sequence analysis of the *GNAS* gene from peripheral blood sample was normal. Lymphocyte activation was normal 3 months post-adrenalectomy, and immunization schedule for live vaccines was established.

### Other findings of MAS

She had breast development and vaginal bleeding that lasted 2 days when she was 7 months old, which repeated five more times after the adrenalectomy till 26 months of age. Breast development was Tanner stage 3, and bone age was markedly advanced (4 years and 2 months), despite severe hypercortisolism. On pelvic ultrasonography, uterus was enlarged to 34×22×24 mm; thus, letrozole (0.625 mg, per oral) was started at 26 months of age.

She also developed marked hypophosphatemia at the age of 6 months (**Table 1**). Radiological investigations since birth demonstrated severe osteopenia and lytic lesions, which were attributed to severe hypercortisolism; however, overt lesions of FD were not confirmed. When she was 9 months old, FGF-23 was elevated [122 pg/ml (normal <52)], which suggested hypophosphatemic rickets associated with FD. Oral phosphate (8 mg/kg) and calcitriol (18 ng/kg) were started. At the age of 23 months, bone survey revealed sclerosis of the base of the skull and maxilla and FD in the lower extremities. She has been on oral phosphate (58.7 mg/kg/day), while calcitriol was ceased.

She is now 34 months old with severe short stature [height, 81 cm (−3.5 SDS); weight, 9,580 g (−3.7SDS)] (**Supplementary Figure S3B**). She had been under regular clinic visits and has been on 15 mg/m^2^/day hydrocortisone and fludrocortisone 0.025 mg/day, letrozole (1×6.25 mg/day), phosphate (58 mg/kg), and ursodeoxycholic acid (100 mg/day) (**Supplementary Figure S2**). She has six words, cannot form two-word sentences, shows body parts, cannot stand up from supine position without support, and takes a few steps with support. Despite regular physiotherapy and ergotherapy, developmental delay is evident (Bayley Scales of Infant and Toddler Development III language scale, 13/79; motor scale, 2/46).

## Discussion

ACTH-independent CS and café-au-lait macules suggested MAS in this case. Interestingly, this patient was admitted for hyponatremia and hyperglycemia requiring insulin treatment. Neonatal MAS and CS are rare conditions, and presentation of this case is quite unique (4).

The earlier the timing of somatic mutation, the greater the burden of G_s_α-mutation-bearing cells leading to widespread tissue involvement in MAS. In the current case, adrenal, hepatic, cardiac, renal, and bone tissue involvement were evident in first weeks of life, while precocious puberty and hypophosphatemic rickets were observed later. A lifetime risk of additional tissue involvement is being acknowledged. CS is the rarest endocrine manifestation of MAS, which appears in <5%–7.1%. It presents exclusively within the first year of life (median age, 3.1 months) where features may develop as early as *in utero* (2–4, 7). The fact that our case was SGA and had moon facies and hirsutism with impaired linear growth, weight gain, hyperglycemia, hypertension, and nephrocalcinosis detected in the neonatal period, suggested severe, *in utero* onset CS. Upon suspicion, both comorbidities (hyperthyroidism, excess growth hormone, FD, and cardiac and hepatobiliary function) of MAS and complications of GC excess (hypertension, hyperglycemia, hyperlipidemia, nephrocalcinosis, decreased bone mineral density, and muscle atrophy) were assessed (1, 3).

Since the initial description of MAS, only 20 neonates with CS have been described with various initial basal serum cortisol ranging from 9.6 to 80.1 µg/dl, and data regarding long-term follow-up and outcome are still developing (1, 2, 8–11). Disease course is heterogenous, and spontaneous resolution of hypercortisolism has been reported (30%) since G_s_-bearing cells are mostly located in the fetal adrenal zone, which normally undergoes apoptosis after birth. However, the outcome is mostly unfavorable in cases with extensive endocrine and extra-endocrine manifestations (1, 2, 8–15). Brown et al. reported poorer prognosis and a lower likelihood of spontaneous remission of adrenal disease in patients with cardiac (cardiomyopathy) and liver involvement (hepatocellular adenomas, inflammatory adenomas, choledochal cysts, neonatal cholestasis, and hepatoblastoma). It was hypothesized that these patients have a greater burden of G_s_α mutation (3, 4).

Treatment of neonatal CS is a long and challenging path where both cortisol excess and its complications should be targeted. Marked hypercortisolism that precipitate neonatal diabetes requiring insulin treatment like our patient is rare and was previously reported only in six patients with CS (4). Until hypercortisolism is managed, hyperglycemia should be treated with insulin. Hypertension is due to mineralocorticoid effect of excess cortisol; thus, blood pressure lowering agents of choice should be aldosterone antagonists (spironolactone) or potassium-sparing diuretics.

The treatment strategy of hypercortisolism is determined by disease severity. In a mildly affected case, medical treatment with an expectation of spontaneous resolution (due to previously stated apoptosis of fetal adrenal zone) may be of choice (3, 4, 16–19). Metyrapone, ketoconazole, and mitotane are medical options for lowering cortisol (20–23). Since our patient had impaired liver function, metyrapone, a potent, rapid acting relatively selective inhibitor of 11-hydroxylase was preferred over ketoconazole for its low risk of hepatotoxicity. Reports reviewing adult data suggest an initial dose of 500–750 mg/day and achievement of biochemical control with 1,500 mg/day (23). However, the initial and maximum dose of metyrapone in neonates is unclear; some authors recommend 300 mg/m^2^/day in four equal doses (6). In our case, adequate biochemical and clinical suppression of cortisol with metyrapone was not achieved despite an increase in dose from 300 to 1,850 mg/m^2^/day.

There are important issues to be considered while using a steroidogenesis inhibitor like metyrapone. Monitoring biochemical response is essential, not only for dose titration and management of cortisol excess but also for adrenal insufficiency due to possible overtreatment. Clinical signs of adrenal insufficiency should always be questioned and assessed. The 24-h urinary free cortisol is the commonly used method; however, it may be impractical due to difficulties in the collection of urine in infants. Alternative methods may be the measurement of early morning serum cortisol and ACTH (23). Low ACTH level may indicate hypercortisolism or may be a sign of suppression due to long-term exposure to hypercortisolism. However, there are deadlocks to be considered in the evaluation of these measurements. A high cortisol level measured by immunoassays does not always indicate an actual elevation. It should be kept in mind that cortisol immunoassays exhibit significant cross-reactivity with cortisol precursors that may be elevated in patients treated with a steroidogenesis inhibitor (especially with metyrapone, which is known to increase 11-deoxycortisol). Such cross-reactivity can be a cause for overestimation of cortisol and may lead to risk of overtreatment (24, 25). It has been suggested that the patients on metyrapone should be biochemically monitored via specific methods, such as mass spectrometry (24–26).

Metyrapone is a relatively selective inhibitor of 11-hydroxylase and 18-hydroxylase. Recent *in vitro* studies indicate greater inhibitory action of metyrapone on aldosterone synthase, resulting in significant reversible reduction in both cortisol and aldosterone. The loss of negative feedback leads to an increase in ACTH, which causes an accumulation of cortisol and aldosterone precursors resulting in an increase in adrenal androgens (23). Although we could not serologically prove an increase in ACTH, hyperpigmentation and the increase in adrenal androgens confirm this mechanism. As far as we know, an increase in DHEA-S causing virilization was an unreported side effect of metyrapone. Clinical (clitoromegaly and hirsutism) and laboratory (DHEA-S) signs of hyperandrogenism should be monitored when higher doses of metyrapone are required.

In the severely affected case with CS, where medical treatment is inadequate and the chance of spontaneous resolution is subsiding, adrenalectomy is indicated when medically feasible. Brown et al. suggested that the presence of comorbid cardiac and liver disease like in our case should prompt consideration for early adrenalectomy (4). Although a previous correlation with initial serum cortisol level and prognosis was not established, it may be speculated that excessively high serum cortisol level is associated with increased number of G_s_α-mutation-bearing adrenal cells. Thus, we suggest that in neonatal CS due to MAS, initial very high serum cortisol levels, like our case, may be a negative prognostic factor both for spontaneous resolution and clinical response to medical treatment. In infants with severe CS, bilateral adrenalectomy is generally performed. Alternatives like unilateral adrenalectomy and one-side total, other-side three-quarters adrenalectomy may be considered to avoid the requirement for lifelong GC and mineralocorticoid replacement. Unilateral adrenalectomy was reported to successfully improve clinical symptoms and endocrinological status in adult studies; nevertheless, recurrence during follow-up was 23.1%, while 17.5% required contralateral adrenalectomy (27–29). Since the causes of CS in adult series are variable and different from pediatric CS due to MAS, it should be borne in mind that reproducibility of adult data is poor. In CS due to MAS, G_s_α-mutation-bearing adrenal gland cells are heterogeneously distributed, and partial adrenalectomy may carry the risk of inadequate management and recurrence. Only a few pediatric case reports addressed this issue. Unilateral adrenalectomy of the larger gland was performed in two neonates with CS due to MAS; remission was achieved for 2 years (30, 31). Itonaga et al. reported a 6-month-old neonate with MAS-associated CS treated with right-sided total adrenalectomy and left-sided half adrenalectomy with remission for 2 years (32). Although these cases were less severe [basal serum cortisol: 16.9, 18.5, and 23.4 µg/dl, respectively (N: 6.2–18.0 µg/dL)], we preferred to perform partial adrenalectomy (right total and left three-quarters adrenalectomy) and succeeded. Our patient has been in remission for more than 2 years.

In the largest case–control analysis of CS in patients with MAS, overall mortality was 20% (six cases) where four of them were deceased following bilateral adrenalectomy (66.7% of all deaths) (4). Anaphylaxis (or adrenal insufficiency), sudden cardiac arrest, sepsis, and sudden death were listed as causes of mortality in those four cases where GC dose and process of GC tapering were not clearly described. The fact that our patient required high-dose GC during peri- and postoperative period to restore well-being, tapering to maintenance dose was very slow, and she is still on maintenance dose GC, suggests that rapid tapering of GCs should be avoided and, although being speculative, may explain sudden death following adrenalectomy.

Gross motor developmental delay may be caused by prenatal exposure to excess GCs. Prenatal GC treatment for possible congenital adrenal hyperplasia or risk of premature birth have been shown to result in cognitive deficits after birth. Furthermore, children who develop CS later in life may experience a decline in cognitive and school performance where the younger the age of onset, the greater the deterioration in IQ scores (3, 4, 33, 34). Since transgenic mice with G_s_α mutation was shown to have short- and long-term memory deficits and impaired associative and spatial learning, it may also be speculated that G_s_α mutation may also be present in the central nervous system (35, 36).

The establishment of diagnosis of FD follows a characteristic and predictable time course. Although *GNAS* mutations are acquired early in embryogenesis, skeletal development appears to be relatively normal *in utero*, without frank clinical signs of FD at birth. Boyce et al. affirmed that FD lesions become apparent over the first several years of life and expand during childhood and adolescence, like our case. Previous case reports have also stated severe osteoporosis, rickets, polyostotic irregular lucencies, pathological fractures, and biopsy-proven FD during infancy (1, 2, 8–15). The exact pathophysiological mechanism is unclear, and G_s_α activation in abnormally differentiated osteocytes is accused. FGF-23 overproduction is an inherent feature of FD, and most patients have elevated circulating levels of FGF-23, but frank hypophosphatemia is rare. The increase in FGF-23 is linked to substantial skeletal involvement. Although FGF-23 levels may wax and wane over time, an increase in FGF-23 usually occurs during periods of rapid growth like infancy and adolescence. Concurrent hyperfunctioning endocrinopathies like hyperthyroidism or CS may also adversely affect bone health.

Peripheral precocious puberty (PP) is the most frequent presenting feature in female patients with MAS (85%) (6). To date, a safe, effective, and long-term treatment for PP in girls with MAS has not been established. The benefits of current interventions on the ultimate outcome of interest, adult height, have not been well-established due to the rarity of the condition and heterogeneous nature of the disease. Despite the small sample size, studies have concluded that letrozole resulted in a statistically significant decrease in the bone age/chronological age ratio, growth velocity, hence increasing predicted adult height (37). Growth outcome in MAS is not only dependent on timing of pubertal onset but on several other disease components (skeletal involvement and endocrinopathies) as well. Hyperthyroidism and growth hormone excess may accelerate growth, while CS may decelerate it (37, 38).

Lack of consensus on both medical and surgical treatment strategies were major obstacles while navigating this case of severe neonatal MAS. The eminence of this report is that it presents current literature with clinical experience on this rare case of neonatal CS due to MAS. High index of suspicion for MAS in a neonate with extensive café-au-lait macules and symptoms of hypercortisolism is the key for early recognition and intervention. Initial excessive cortisol in neonatal CS may be a negative prognostic factor for spontaneous resolution and response to medical treatment, indicating early right total and left three-quarters adrenalectomy. Post-adrenalectomy survival may be related to close supervision during GC tapering.

## Data availability statement

The datasets presented in this study can be found in online repositories. The names of the repository/repositories and accession number(s) can be found in the article/**Supplementary Material**.

## Ethics statement

Written informed consent was obtained from the individual(s), and minor(s)’ legal guardian/next of kin, for the publication of any potentially identifiable images or data included in this article.

## Author contributions

YU collected and analyzed data, drafted the initial manuscript, and reviewed and revised the manuscript. OG collected data. İU, HH, BG, SE, and TK collected data and reviewed and revised the manuscript. ZO and EG analyzed data, conceptualized the work, and revised and critically reviewed the manuscript for important intellectual and medical content. All authors approved the final manuscript as submitted and agree to be accountable for all aspects of the work.

## Supplementary material

Supplementary Figure 1
**(A)** The findings of physical and radiologic examination. Notice cushingoid facies, hyperpigmented macules that does not cross the midline at the front of the trunk. **(B)** Anteroposterior radiographs reveal irregularities in radius, ulna and femur. Although generalized osteopenia improves at 34 months, FD lesions become prominent over months.

Supplementary Figure 2 Timeline of the course of symptoms in neonatal McCune Albright Syndrome noting adjustments made in treatment. Grey box denotes age in days for the first month of life then in months. NPH: Neutral Protamine Hagedorn insulin, CS: Cushing syndrome, PP: precocious puberty.

Supplementary Figure 3
**(A)** Change in serum cortisol with increased metyrapone (methyrapone was initiated on day 25). **(B)** Growth chart, the arrow represents right total and left three quarters adrenalectomy.

Supplementary Figure 4 Representative histological features of nodular adrenal hyperplasia. **(A, B)** show low-power while **(C)** Show high-power views.
