# Supplementary figures and images for: Case Report: Severe McCune–Albright syndrome presenting with neonatal Cushing syndrome: navigating through clinical obstacles
Source: Front Endocrinol (Lausanne). 2023 Jul 25;14:1209189. doi: 10.3389/fendo.2023.1209189 (PMC10407558; doi:10.3389/fendo.2023.1209189)

|  | Neonate | 4 months | 34 months (postadrenalectomy) |
|--|---------|----------|-------------------------------|
|--|---------|----------|-------------------------------|

**A**

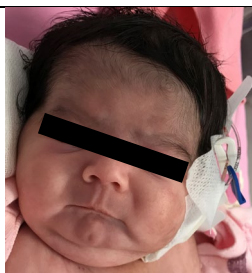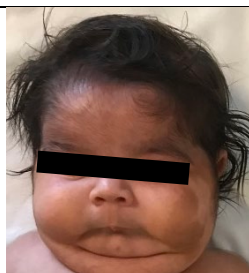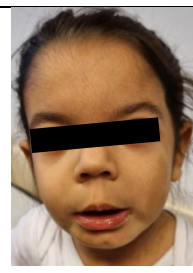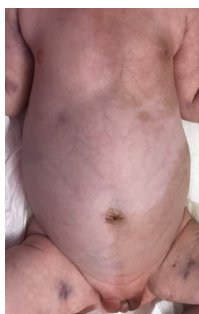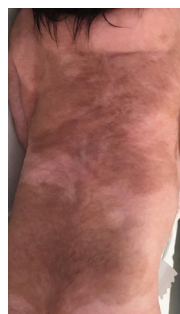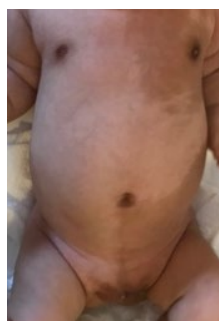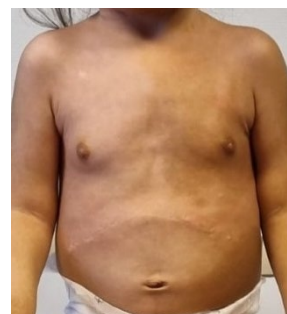

**B**

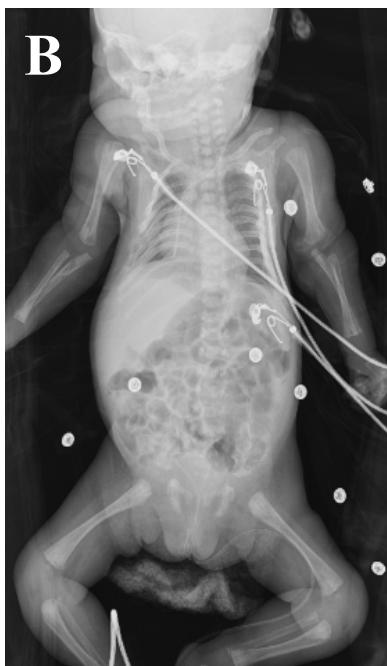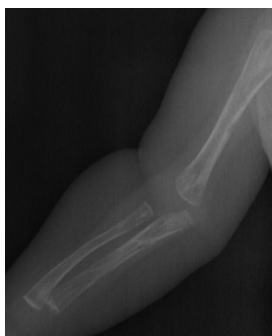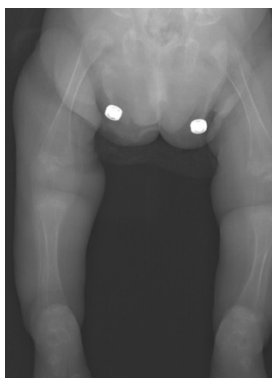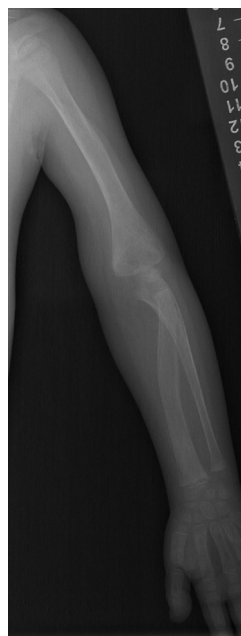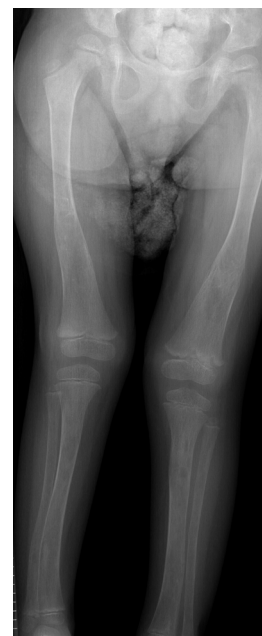

Supplement: Supplementary Figure 1 — (A) The findings of physical and radiologic examination. Notice cushingoid facies, hyperpigmented macules that does not cross the midline at the front of the trunk. (B) Anteroposterior radiographs reveal irregularities in radius, ulna and femur. Although generalized osteopenia improves at 34 months, FD lesions become prominent over months. [file Image_1.pdf]

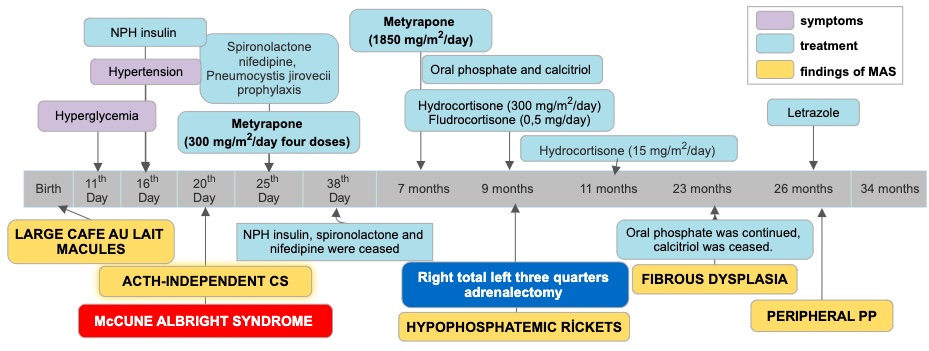

Supplement: Supplementary Figure 2 — Timeline of the course of symptoms in neonatal McCune Albright Syndrome noting adjustments made in treatment. Grey box denotes age in days for the first month of life then in months. NPH: Neutral Protamine Hagedorn insulin, CS: Cushing syndrome, PP: precocious puberty. [file Image_2.jpeg]

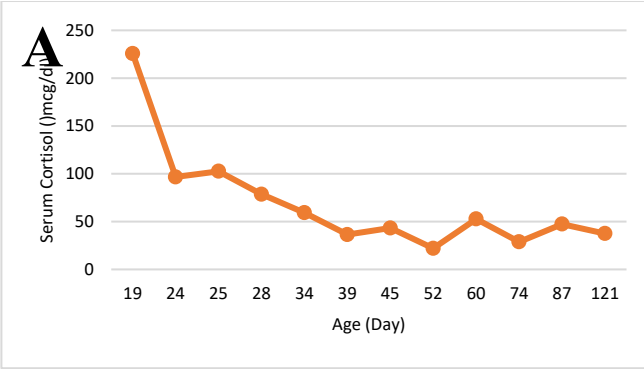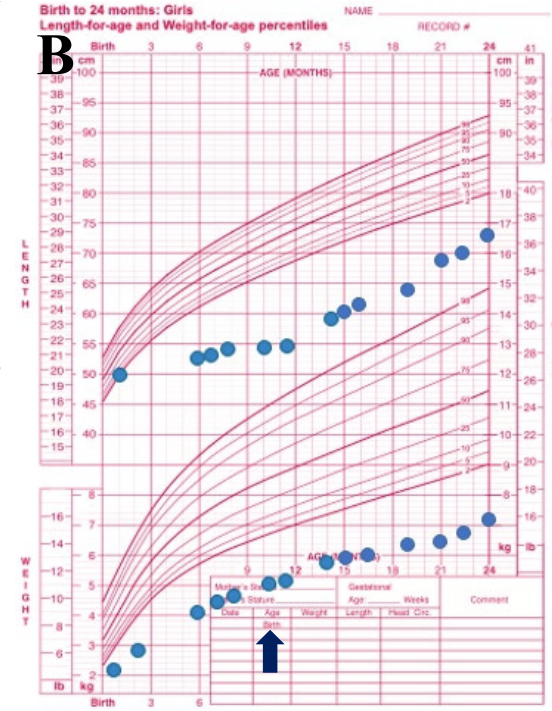

Supplement: Supplementary Figure 3 — (A) Change in serum cortisol with increased metyrapone (methyrapone was initiated on day 25). (B) Growth chart, the arrow represents right total and left three quarters adrenalectomy. [file Image_3.pdf]

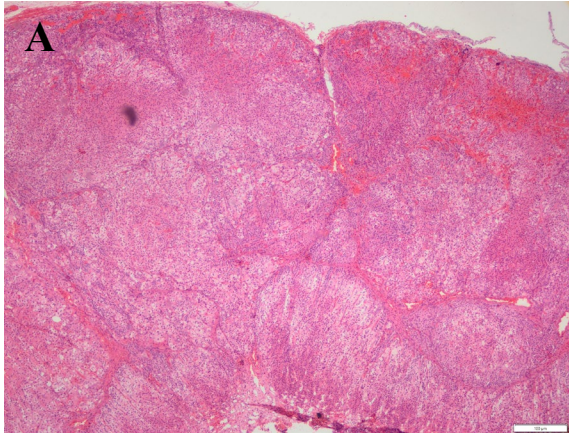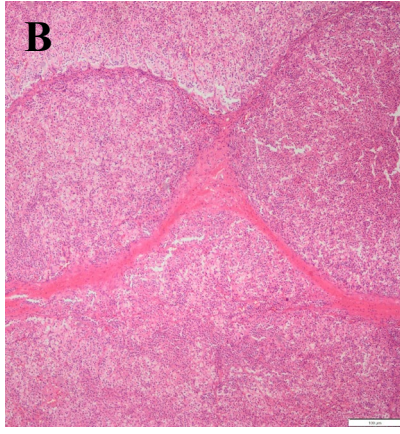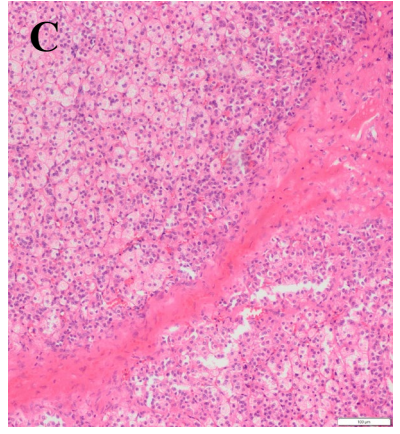

Supplement: Supplementary Figure 4 — Representative histological features of nodular adrenal hyperplasia. (A, B) show low-power while (C) Show high-power views. [file Image_4.pdf]
